# Epidemiological profile of patients with end stage renal disease in a referral hospital in Cameroon

**DOI:** 10.1186/s12882-015-0044-2

**Published:** 2015-04-21

**Authors:** Marie P Halle, Christian Takongue, Andre P Kengne, François F Kaze, Kathleen B Ngu

**Affiliations:** 1 Department of clinical sciences, Faculty of medicine and pharmaceutical science, University of Douala, Douala, Cameroon; 2 Department of Internal Medicine, Douala General Hospital, Douala, Cameroon; 3 South African Medical Research Council and University of Cape Town, Cape Town, South Africa; 4 Department of internal medicine and specialties, Faculty of medicine and biomedical sciences, University of Yaounde I, Yaounde, Cameroon

## Abstract

**Background:**

Data regarding the epidemiology of end-stage renal disease (ESRD) and dialysis in sub-Saharan Africa are scarce and knowledge about the spectrum renal disease is very limited. This study is on the profile of patients with ESRD in a referral hospital in Cameroon.

**Methods:**

Medical records of patients with ESRD covering a 10-year period of activities of the Douala General Hospital were reviewed. Data were retrieved on socio demographic, and clinical data such as major comorbidities, the presumed aetiology of ESRD, blood pressure, biological variables and renal replacement therapy.

**Results:**

In all 863 patients were included with 66% being men. Mean age was 47.4 years overall, 48.9 in men and 44.5 in women (p < 0.001). The main background aetiologies of ESRD were hypertension (30.9%), glomerulonephritis (15.8%), diabetes (15.9%), HIV (6.6%) and unknown (14.7%). Participants with HIV, glomerulonephritis or unknown background nephropathy were younger, more likely to be women, to be single and unemployed, while those with hypertension and/or diabetes were older, more likely to be men, to be either married or widow, and to be retired or working in the public sector. A total of 677 patients started haemodialysis with decreasing trend across age quartiles (p = 009), and variation across background nephropathies (p < 0.001). Emergency dialysis unplanned on a temporary catheter occurs in 88.3% of patients.

**Conclusion:**

This study has revealed substantial gender and age differentials in the socio-demographic features and background nephropathy in patients with ESRD in this setting. The likelihood of starting maintenance dialysis varied across background nephropathies, driven at least in part by age differences across background nephropathies.

## Background

End-stage renal disease (ESRD) is a major public health problem worldwide and is associated with considerable morbidity and mortality [1,2]. It has been estimated that the prevalence of ESRD will rise over the next decades, driven by population aging, and increasing prevalence of diabetes mellitus and hypertension [3,4]. This will occur predominantly in developing countries [5-8], such as those in sub-Saharan Africa (SSA), where poverty is rampant [9,10]. In addition to non-communicable diseases, communicable diseases especially infections (HIV, hepatitis) are common causes of CKD in Africa. Therefore the epidemiology of ESRD is likely different in SSA, with people affected at a much younger age [11-17]. Although data are increasingly emerging to characterize the burden of CKD in SSA [18-20], the profile of ERSD which is the final common pathway for CKD, has yet to be optimally described in most SSA settings. In Cameroon, renal replacement therapy started in the early 1980, initially in the Capital city (Yaounde), then was subsequently extended to the economic capital (Douala), and more recently (in 2008) to few other cities in the country [21]. However, just likes in most other SSA countries, nephrology services in Cameroon are not widely available and are mostly understaffed. In 2012 for instance, the country had a total of five nephrologists who were all practicing in the two main cities of the country. In this same year the country had eight hemodialysis centers providing care to about 500 patients with ESRD. Since the year 2002, dialysis services are highly subsidized in public centers [21]. One collateral effect of these measures has been the increasing number of people with various stages of CKD reporting to or referred to public institutions with dialysis facilities. This provide the opportunity of characterizing some specific segments of the populations with CKD, in the absence of large community based-studies. Accordingly, the main purpose of this study was to describe the profile of patients with ESRD in Cameroon, using data from one of the major referral hospital for ESRD in the country. Such information is needed for health service planning and policy formulation.

## Methods

### Study setting

The study was carried out in the renal unit of the Douala general hospital (DGH) in Cameroon. DGH is tertiary referral hospital with a capacity of 320 beds. It has the largest haemodialysis unit of the country, and serves as referral hospital for patients with kidney disease in the Littoral region of the country (approximately 3 million population in 2012) and beyond. Patients with kidney disease referred to the unit are assigned a unique identifier for the follow-up purpose. At the first consultation in the unit and at dialysis initiation each patient has clinical assessment and laboratory tests done. The center has always functioned with one nephrologist and one general practitioner except the period between 2005 and 2007 (2 nephrologists one general practitioner). At the end of the year 2012, the unit was operating with one nephrologist, two general practitioners and 12 nurses. The center was equipped with 17 HD Fresenius® 4008S generators (Fresenius Medical Care, Hamburg, Germany), used synthetic polysulfone dialysis membrane and bicarbonate dialysate, and provided dialysis to 140 patients. All patients underwent 2 dialysis sessions of 4 hour per week. The study received administrative authorization from the DGH and ethical approval was obtained from the Douala University Ethics Committee. The study was based on routinely collected data; accordingly individual informed consent to participate does not apply.

### Patients and methods

This study was based on medical files of patients with ESRD seen in the unit from January 2002 to December 2012. Of 1009 files retrieved 94 (9.3%) were excluded for acute renal failure and 52 (5.1%) with ESRD for missing data on kidney function. Therefore 863 patients (85.5% of the starting sample) were included in the final analysis. For all patients the following data were extracted: socio-demographic including age (in years), sex, profession, marital status, and clinical data such as major comorbidities (hypertension, diabetes, HIV, gout, history of stroke) the presumed aetiology of ESRD, blood pressure, biological variables, vascular access at dialysis initiation, effective start of RRT, and reason for non-initiation of dialysis.

### Operational definitions

The diagnosis of ESRD was based on the following: estimated glomerular filtration rate (eGFR) based on the Cockcroft and Gault formula (from 2002 to 2010) [22] or the four-variable Modification of Diet in Renal Disease (MDRD) formula (from 2010 and beyond) [23] lower than 15 ml/min/1.73 m^2^, bilateral shrunken kidneys on ultrasound and the presence of any of the following clinical or biological signs of uraemia (anaemia, asthenia, anorexia, vomiting, muscles cramps, hiccup, hypocalcaemia and hyperphosphoremia) and need for chronic dialysis. Serum creatinine measurements to estimate GFR used a kinetic modification of the Jaffé reaction, with conversion to standardized values before implementation in the MDRD equation as appropriate [24,25]. Hypertension, diabetes and HIV were based on documented history, ongoing drug treatments or a documented systolic (and/or diastolic) blood pressure >140 (90) mmHg for hypertension or fasting blood glucose >126 mg/dl, or a positive test for HIV at baseline. The aetiology of kidney disease was mostly based on clinical arguments in these patients presenting late with CKD and in the context of unavailability and/or unaffordability of renal histology. Chronic glomerulonephritis was based either on a past history of a documented glomerular disease or the presence of a glomerular syndrome (proteinuria and/or haematuria, hypertension in the absence of identifiable secondary causes). Background nephropathy was ascribed to HIV in the presence of a glomerular syndrome (nephrotic range proteinuria, and/or haematuria) in an HIV positive patient with hyperechogenic and normal size kidneys on ultrasound, and in the absence of any other secondary cause. Emergency dialysis was defined as dialysis initiation unplanned in a life threatening situation (such as pulmonary oedema, uremic encephalopathy, and severe hyperkalaemia) on a temporary vascular access (non tunnelled polyureththane double lumen central venous catheter). The Charlson comorbidity score was estimated adding up the scores assigned to existing comorbidities based on the risk of dying associated with each of them. The following comorbidities were considered: congestive heart failure, coronary disease, diabetes mellitus, malignancies, stroke, peripheral vascular disease, chronic obstructive pulmonary disease, HIV/AIDS, chronic liver disease and connective tissue disease [26].

### Statistical analysis

Data were analysed with the use of Statistical Package for Social Sciences SPSS® v.17 for Windows. We have presented the results as count and percentages, mean and standard deviation (SD). Groups’ comparisons used the Student’s *t*-test, the analysis of the variance (ANOVA), Mann–Whitney *U* test and Kruskal-Wallis tests for continuous variables and chi-square tests and equivalents for categorical variables. The Cochran-Armitage trend test and Brown-Forsythe Levene procedures were used to test the linearity of the trends across quartiles of age. The level of significance was set at p <0.05.

## Results

### Profile of participants overall and by gender

Eight hundred and sixty three patients were included with 66% being men. The baseline characteristics of patients are shown in Table 1. Mean age was 47.4 years overall, 48.9 in men and 44.5 in women (p < 0.001). In all 71.9% (n = 542) of patients were married and the distribution of marital status was different between men and women (p < 0.001). In all 33.1% (n = 250) were unemployed, 24.6% (n = 186) were employed in the private sector, 26.2% (n = 198) in the public sectors, while 16% (n = 121) were retired. The employment status was significantly different by gender (p < 0.001) with more men working in the public and private sector, or being employed while more women were unemployed. At baseline 146 (16.9%) participants had history and/or current heart disease, similarly in men and women (p = 0.701); 20 (2.3%) had a history of stroke (p = 0.235 for men vs. women); 69 (8.0%) had a positive status for HIV infection (p = 0.086); 14 (1.6%) had active tuberculosis (p = 0.157); while 12 (1.4) had diagnosed cancer (p = 0.120). Furthermore 763 (88.4%) had hypertension, similarly in men and women (p = 0.654); 245 (28.4%) had diabetes mellitus (men vs. women: 32.1% vs. 23.2%; p = 0.017); while 142 (16.5%) were alcohol drinker en 65 (7.5%) smokers, with always higher prevalence in men than in women (both p ≤ 0.002) The main background aetiologies of ESRD were hypertension (30.9%, n = 267), chronic glomerulonephritis (15.8%, n = 136), diabetes (15.9%, n = 137) and HIV (6.6%, n = 57). No aetiology was identified in 14.7% (n = 127) of the participants and 2.7% had a polycystic kidney disease. The distribution of background aetiologies of CKD was significantly different in men and women (p = 0.021), with more men having hypertension, diabetes mellitus, while more women had glomerulonephritis and HIV (Table 1). With the exception of haemoglobin levels (p = 0.004) and urea levels (p = 0.015), the biological profile was generally similar in men and women.

**Table 1:** **Profile of patients with ESRD overall over 10 years of the study**

Variables	Overall	Male	Female	p-value	Quartiles of age				
					Q1	Q2	Q3	Q4	p-trend
N (%)	863 (100)	570 (66.0)	293 (34.0)		205 (23.8)	218 (25.3)	221 (25.6)	219 (25.4)	
Marital status, n (%)				<0.001					<0.001
Single	169 (22.4)	93 (18.7)	76 (29.6)		120 (67.4)	31 (16.5)	14 (7.0)	4 (2.1)	
Married	542 (71.9)	393 (79.1)	149 (58.0)		55 (30.9)	147 (27.1)	169 (84.9)	171 (90.5)	
Divorced	3 (0.4)	1 (0.2)	2 (0.8)		0	1 (0.5)	1 (0.5)	1 (0.5)	
Widow	40 (5.3)	10 (2.0)	30 (11.7)		3 (1.7)	9 (4.8)	15 (7.5)	13 (6.9)	
Employment status, n (%)				<0.001					<0.001
No employment	250 (33.1)	74 (15.8)	176 (67.5)		55 (30.2)	1 (0.5)	1 (0.5)	1 (0.5)	
Private sector	186 (24.6)	147 (28.9)	39 (14.9)		48 (26.4)	71 (37.2)	44 (22.9)	23 (12.1)	
Public sector	198 (26.2)	163 (33.0)	35 (13.4)		25 (13.7)	71 (37.2)	74 (38.5)	28 (14.7)	
Retired	121 (16.0)	110 (22.3)	11 (4.2)		1 (0.5)	1 (0.5)	29 (15.1)	90 (47.4)	
Mean age, yr. (SD)	47.4 (14.8)	48.8 (14.3)	44.5 (15.5)	<0.001	26.3 (7.5)	43.8 (3.5)	53.3 (2.3)	64.6 (6.1)	<0.001
Age, min-max, years	7.0-87.0	11.0-87.0	7.0-79.0		7.0-37.0	38.0-49.3	50.0-57.0	58.0-87.0	
Age, median [25th-75th percentiles]	50 [38–58]	51 [41–58]	46 [32–56]		27 [20-32]	44 [41–47]	53 [51–55]	63 [60–68]	
Any hypertension, n (%)	763 (88.4)	506 (88.8)	257 (87.7)	0.654	170 (82.9)	180 (82.6)	209 (94.6)	204 (93.2)	<0.001
Any heart disease, n (%)	146 (16.9)	99 (17.4)	47 (16.0)	0.701	16 (7.8)	42 (19.3)	32 (14.5)	56 (25.6)	<0.001
Diabetes, n (%)	245 (28.4)	177 (31.1)	68 (23.2)	0.017	9 (4.4)	38 (17.4)	102 (46.2)	96 (43.8)	<0.001
HIV positive, n (%)	69 (8.0)	39 (6.8)	30 (10.2)	0.086	23 (11.2)	24 (11.0)	16 (7.2)	6 (2.7)	<0.001
Alcohol drinker, n (%)	142 (16.5)	113 (19.8)	29 (9.9)	<0.001	15 (7.3)	39 (17.9)	55 (24.9)	33 (15.1)	0.010
Smoking, n (%)	65 (7.5)	54 (9.5)	11 (3.8)	0.002	5 (2.4)	19 (8.7)	30 (13.6)	11 (5.0)	0.137
Cerebrovascular accidents, n (%)	20 (2.3)	16 (2.8)	4 (1.4)	0.235	1 (0.5)	4 (1.8)	9 (4.1)	6 (2.7)	0.053
Cancer, n (%)	12 (1.4)	5 (0.9)	7 (2.4)	0.120	2 (1.0)	5 (2.3)	3 (1.4)	2 (0.9)	0.731
Tuberculosis, n (%)	14 (1.6)	12 (2.1)	2 (0.7)	0.157	5 (2.4)	4 (1.8)	4 (1.8)	1 (0.5)	0.123
Mean Hb, g/dl (SD)	7.7 (1.9)	7.9 (2.0)	7.4 (1.8)	0.004	7.3 (1.8)	7.7 (2.1)	8.1 (1.9)	7.8 (1.8)	0.004
Mean Urea, mg/l (SD)	2.4 (1.1)	2.5 (1.2)	2.2 (1.0)	0.015	2.7 (1.1)	2.6 (1.2)	2.2 (1.0)	2.2 (1.1)	<0.001
Mean creatinine,mg/l (SD)	171.3 (88.5)	175.8 (92.5)	162.6 (79.9)	0.064	198.0 (95.4)	185.9 (91.7)	160.9 (77.9)	142.4 (78.6)	<0.001
Mean sodium, mmol/l (SD)	136.8 (11.3)	136.3 (11.6)	137.6 (10.5)	0.208	137.3 (10.8)	135.9 (13.5)	137.4 (8.9)	136.5 (11.4)	0.875
Mean potassium, mmol/l (SD)	5.3 (1.2)	5.3 (1.2)	5.3 (1.2)	0.774	5.3 (1.2)	5.4 (1.3)	5.2 (1.1)	5.3 (1.1)	0.890
Mean chlorine,mmol/l (SD)	100.8 (13.7)	100.4 (14.3)	101.6 (12.4)	0.347	101.2 (8.1)	100.3 (14.8)	100.6 (15.5)	101.2 (14.7)	0.938
Mean Ca2+,mg/l (SD)	80.0 (14.6)	80.1 (15.2)	80.0 (13.4)	0.842	75.6 (17.0)	81.0 (13.0)	79.7 (12.5)	83.7 (12.9)	0.001
Mean phosphate,mg/l (SD)	70.5 (37.4)	70.2 (32.9)	71.1 (45.0)	0.829	73.0 (36.1)	73.9 (42.5)	70.1 (29.5)	65.6 (40.9)	0.119
Dialysis start, n (%)	677 (78.4)	449 (78.8)	228 (77.8)	0.746	173 (84.4)	178 (81.7)	168 (76.0)	158 (72.1)	0.009
Background nephropathy, n (%)				0.021					<0.001
Hypertension	267 (30.9)	190 (33.3)	77 (26.3)		26 (12.7)	86 (39.4)	75 (33.9)	80 (36.6)	
Diabetes	137 (15.9)	100 (17.5)	37 (12.6)		5 (2.4)	20 (9.2)	63 (28.5)	49 (22.4)	
Hypertension and diabetes	65 (7.5)	44 (7.7)	21 (7.2)		4 (2.0)	5 (2.3)	24 (10.9)	32 (14.6)	
Chronic glomerulonephritis	136 (15.8)	82 (14.4)	54 (18.4)		98 (47.8)	22 (10.1)	10 (4.5)	6 (2.7)	
HIV	57 (6.6)	30 (5.3)	27 (9.2)		21 (10.2)	20 (9.2)	11 (5.0)	5 (2.3)	
Others	74 (8.6)	48 (8.4)	26 (8.9)		12 (5.9)	20 (9.2)	21 (9.5)	21 (9.6)	
Unknown	127 (14.7)	76 (13.3)	51 (17.4)		39 (19.0)	45 (20.6)	17 (7.7)	26 (11.9)	
Median Charlson Score (Q1-Q3)	3 [2-4]	3 [2-4]	3 [2-4]	0.312	2 [2]	2 [2]	3 [3]	4 [4,5]	<0.001

### Profile of participants across age quartiles

Being married or widow increased across increasing age quartiles while being single decreased (p < 0.001 for linear trend). Mean haemoglobin (p = 0.004), creatinine (p < 0.001), calcium (p = 0.001) and Charlson score (p < 0.001) linearly increased with age while urea (p < 0.001) and likelihood of starting dialysis (p = 0.009) linearly decreased from 84.4% (173/205) in the lower age quartile to 72.1% (158/219) in the upper quartile. History of cerebrovascular accident (p = 0.053), cancer (p = 0.731), tuberculosis (p = 0.123) and smoking (p = 0.137) were mostly non-linear across age quarters while other risk factors and co-morbidities varied in a linear fashion with age (all p ≤ 0.01 for linear trend). Hypertension, glomerulonephritis, HIV infection and unknown aetiology for the background nephropathy decreased with increasing age while diabetes alone or with hypertension increased (p < 0.001, Table 1).

### Profile of participants across background nephropathies

Participants with HIV, glomerulonephritis or unknown background nephropathy were younger, more likely to be women, to be single and unemployed, while those hypertension, diabetes alone or with hypertension were older (p < 0.001), more likely to be men (p = 0.021), to be either married or widow (p < 0.001), and to be retired or working in the public sector (p < 0.001). In general risk factors and comorbidities varied significantly across background nephropathies (all p ≤ 0.025), with the exception of cerebrovascular diseases (p = 0.089). There was also significant differences across background nephropathy subgroups for haemoglobin (p < 0.001), urea (p < 0.001), creatinine (p < 0.001) phosphate (p = 0.024) and Charlson score (p < 0.001), Table 2.

**Table 2:** **Profile of patients with ESRD by background nephropathy**

Variables	Hypertension	Diabetes	Diabetes & HTN	Glomerulonephritis	HIV	Others	Unknown	p-value
N (%)	267 (30.9)	137 (15.9)	65 (7.5)	136 (15.8)	57 (6.6)	74 (8.6)	127 (14.7)	NA
Men, n (%)	190 (71.2)	100 (73.0)	44 (67.7)	82 (60.3)	30 (52.6)	48 (64.9)	76 (59.8)	0.021
Marital status								<0.001
Single	25 (10.9)	7 (5.7)	6 (10.5)	79 (59.8)	15 (27.8)	9 (14.1)	28 (29.5)	
Married	193 (83.9)	110 (90.2)	44 (77.2)	49 (37.1)	31 (57.4)	52 (81.3)	63 (66.3)	
Divorced	1 (0.1)	0	0	1 (0.8)	0	1 (1.6)	0	
Widow	11 (1.5)	5 (4.1)	7 (12.3)	3 (2.3)	8 (1.1)	2 (14.8)	4 (4.2)	
Employment status								<0.001
No employment	52 (22.8)	27 (21.8)	14 (25.4)	80 (60.1)	16 (32)	17 (26.2)	44 (44)	
Private sector	61 (26.8)	16 (12.9)	7 (12.7)	34 (25.6)	17 (34.0)	18 (27.7)	33 (33.0)	
Public sector	74 (32.5)	45 (36.3)	16 (29.1)	17 (12.8)	15 (30.0)	14 (21.5)	17 (17.0)	
Retired	41 (18.0)	36 (29.0)	18 (32.7)	2 (1.5)	2 (4.0)	16 (24.6)	6 (6.0)	
Mean age, yr. (SD)	51.8 (11.9)	55.5 (10.1)	56.9 (10.5)	30.4 (13.5)	42.3 (10.4)	49.7 (12.1)	43.4 (14.4)	<0.001
Any hypertension, n (%)	265 (99.3)	133 (97.1)	65 (100)	130 (95.6)	38 (66.7)	52 (10.3)	80 (63.0)	<0.001
Any heart disease, n (%)	49 (18.4)	32 (23.4)	13 (20.0)	13 (9.6)	6 (10.5)	8 (10.8)	25 (19.7)	0.025
Diabetes, n (%)	19 (7.1)	135 (98.5)	64 (98.5)	6 (4.4)	6 (10.5)	7 (9.5)	8 (6.3)	<0.001
HIV positive, n (%)	2 (0.7)	2 (1.5)	2 (3.1)	1 (0.7)	57 (100)	2 (2.7)	3 (2.4)	<0.001
Alcohol drinker, n (%)	48 (18.0)	27 (19.7)	22 (33.8)	13 (9.6)	6 (10.5)	10 (13.5)	16 (12.6)	0.001
Smoking, n (%)	49 (18.4)	32 (23.4)	13 (20.0)	13 (9.6)	6 (10.5)	8 (10.8)	25 (19.7)	0.025
Cerebrovascular accidents, n (%)	9 (3.4)	3 (2.2)	4 (6.2)	2 (1.5)	1 (1.8)	1 (1.4)	0	0.089
Cancer, n (%)	2 (0.7)	0	1 (0.5)	0	0	7 (9.5)	2 (1.6)	<0.001
Tuberculosis, n (%)	1 (0.4)	3 (2.2)	2 (3.1)	1 (0.7)	6 (10.5)	1 (1.4)	0	<0.001
Mean Hb, g/dl (SD)	7.8 (2.0)	8.4 (1.8)	7.7 (1.8)	7.2 (1.8)	7.6 (1.6)	7.7 (2.0)	7.3 (1.9)	<0.001
Mean Urea, mg/l(SD)	2.4 (1.1)	1.9 (0.9)	2.2 (1.1)	2.2 (1.1)	2.6 (1.2)	2.3 (1.0)	2.9 (1.3)	<0.001
Mean creatinine, mg/l (SD)	173.5 (88.5)	121.5 (62.5)	134.5 (50.8)	186.5 (83.0)	185.9 (84.5)	163.0 (83.4)	222.1 (105.3)	<0.001
Mean sodium,mmol/l (SD)	136.9 (9.6)	136.7 (13.8)	140.1 (8.6)	137.1 (8.6)	135.6 (11.7)	134.0 (19.0)	136.1 (8.4)	0.217
Mean potassium, mmol/l (SD)	5.2 (1.3)	5.3 (1.0)	5.2 (1.2)	5.4 (1.3)	5.2 (1.2)	5.6 (1.0)	5.3 (1.1)	0.506
Mean chlorine, mmol/l (SD)	99.7 (13.9)	102.0 (13.8)	101.5 (21.7)	99.9 (12.9)	99.8 (9.1)	105.0 (11.9)	100.4 (10.4)	0.383
Mean Ca2+,mg/l (SD)	80.6 (13.3)	83.9 (12.0)	81.3 (12.5)	77.9 (15.2)	77.0 (12.9)	81.4 (12.5)	78.0 (18.0)	0.268
Mean phosphate,mg/l (SD)	73.1 (37.2)	56.1 (28.2)	71.2 (28.2)	71.9 (34.1)	76.5 (36.0)	70.5 (35.7)	79.3 (55.9)	0.024
Dialysis start, n (%)	194 (72.7)	94 (68.6)	50 (76.9)	116 (85.3)	44 (77.2)	63 (85.1)	116 (91.3)	<0.001
Median Charlson Score (Q1-Q3)	3 [2,3]	3 [3,4]	3 [3,4]	2 [2]	6 [6,7]	3 [2-6]	2 [2,3]	<0.001

### Dialysis start and variation across major subgroups

**Figure 1:**
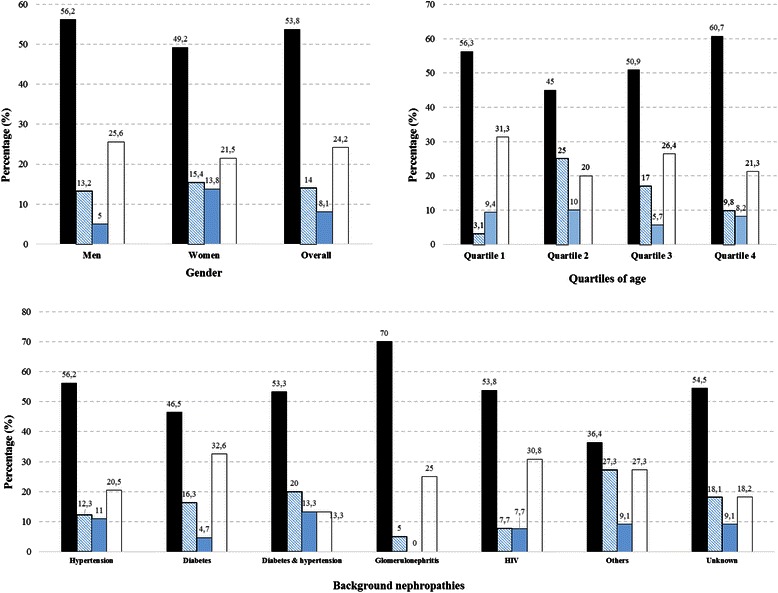
Reasons for not starting dialysis in major subgroups. Figure panels are for major subgroups defined by gender (upper left panel), quartiles of age (upper right panel) and background nephropathy (lower panel). Colour codes representing the different reasons for not starting dialysis are for financial reasons alone (black), Fear of dialysis alone (diagonal blue accent lines), financial reasons and fear of dialysis (blue accent) and unknown reasons (white). The broken horizontal lines have been added to assist visual interpretation of the figures. For each figure panel, the number at the top of each bar are for the percentage of the corresponding reason within the subgroup. The p-value for the comparison of the relevant subgroups are 0.175 for gender, 0.172 for quartiles of age and 0.735 for background nephropathy.

A total of 677 (78.4%) patients started haemodialysis with no difference by sex (p = 0.746) but a significant linearly decreasing trend across age quartiles (p = 009), Table 1; and a significant variation across background nephropathies (p < 0.001, Table 2). Variations in the dialysis uptake across years was not significant (p = 0,246) and did not follow a linear trend (p = 0,648 for linear trend). The least dialysis initiation was observed in patients with diabetes (68.6%), followed by those with hypertension (72.7%) and those with diabetes and hypertension (76.9%) while the highest dialysis uptake was in patients with unknown background nephropathy (91.3%). Reasons for not starting dialysis among 181 (21.5%) participants were financial constraints (53.8%), fear of dialysis (14%), or the combination of both (8.1%), while the reason was unknown in 24.2%. Reasons for not starting dialysis are depicted in Figure 1 overall and by major subgroups showing no difference between men and women (p = 0.175), across age quartiles (p = 0.272) and across background nephropathies (p = 0.735). Emergency dialysis unplanned on a temporary catheter occurs in 88.3% of patients.

## Discussion

Based on a large sample of patients treated in a tertiary referral hospital, the current study provides information on the profile of patients with ESRD in Cameroon. These patients comprise mostly middle age men. One third of the participants and particularly women and young patients were unemployed. Hypertension, diabetes, chronic glomerulonephritis and to some extend HIV were the leading cause of ESRD. Across background nephropathies, participants with HIV, glomerulonephritis or unknown aetiology were younger, more likely to be women, to be single and unemployed, while those with hypertension, diabetes alone or with hypertension were older, more likely to be men, to be married or widowed, and to be retired or employed. They present as expected with abnormal biological profile, similarly in men and women (with the exception of haemoglobin and urea levels) but with variation by background nephropathy. Charlson comorbidity score increased with age and background nephropathy with HIV patients having the highest score. One in five patients eligible for dialysis did not start such a treatment, while among those who started, the rate of emergency dialysis on a temporary catheter was very high.

In this study patients were relatively young, compared with reports from developed countries were ESRD affects more elderly people [27]; but our results are in agreement with many reports from developing countries [11-17,28]. This young age can be explain at least in part by differences in the etiologic profile of CKD, with conditions of young age such as glomerulonephritis and HIV infections playing an important part in our setting. Early occurrence in Blacks of some major risk factors like hypertension, the low awareness, detection, treatment and control of blood pressure likely contribute as well [19,29-32]. But these may not explain the male dominance among those with CKD in our setting. It is however known that the male sex is a risk factor for CKD and the male predominance among the ESRD population is a worldwide phenomenon [33,34].

Based on clinical assessment alone, the finding in this study of hypertension being the leading background nephropathy, followed by chronic glomerulonephritis and diabetes is consistent with previous studies from SSA. [17,35,36]. Even if hypertensive nephrosclerosis is a more prevalent in blacks in the absence of histology studies, it is unclear what proportion of hypertension in our sample could be secondary to a primary renal disease. The high proportion of ESRD from HIV nephropathy in women and young people mirrors the demographic characteristics of HIV infection in SSA [37,38].

Financial constraints is a known cause for not receiving RRT in developing countries [39,40]. In this study one in five participants could not start dialysis mainly due to financial reasons. In Cameroon there is no social insurance and most patients have to pay out of pocket for their health cost. Despite the high state subsidies for dialysis session (95%), the cost of out of pocket expenditure for haemodialysis remain high and not affordable for most patients. The lack of awareness of the importance of RRT and the absence of psychological preparation could explained the fear of dialysis. The need for early detection and preventative measures with regard to CKD in our area is obvious. The proportion of patients starting HD unplanned, unprepared on a temporary access in this study was among the highest reported so far. This is mainly due to late presentation or referral of patients to the nephrologist as it is already known in the study setting.^13^ The adverse outcomes of unplanned dialysis are well known and include increase morbidity, poor quality of life and increased mortality [41-43].

### Study strengths and limitations

The limitations of research based on health records have been largely characterized, and apply for many to the current study. It is however of note that there has been effort to systematically collect in standardized fashions, core data from patients with CKD and or commencing dialysis in this setting, which limits the potentially effects of missing data, and make data collection consistent across years. The background nephropathy in this patients largely reporting with advanced CKD was mostly based on clinical arguments. Such an approach may not be all accurate considering for instance that the differentiation between hypertension causing ESRD and hypertension resulting from or co-occurring with ESRD may not be possible from a cross-sectional analysis of patients with ESRD. The Cockcroft-Gault (CG) formula and MDRD equation were used at different time-points to estimate kidney function and confirm the diagnosis of ESRD. Compared with MDRD (which is more accurate), CG tends to overestimate kidney function, which in turn could falsely lead to the classification of some patients with ESRD as not having the condition. Such misclassification however will have no effect on our findings and conclusions of the current study. A major strength of the current study is the large sample size which has allowed us to generate stable estimates and make robust comparisons. Furthermore, the study center remains the facility with the largest haemodialysis unit in the country and has for most of the study period been one of the two public hospitals with dialysis facility. It is therefore very likely that the population described in the current study is representative of the population of people with ERSD in the country.

## Conclusion

ESRD patients in Cameroon are relatively young and comprised most men. Hypertension and diabetes were the leading cause of ESRD, affecting more men and older patients, while chronic glomerulonephritis and HIV were common in women and young patients. A considerable number of patients could not start dialysis mainly for financial constraint in a setting where no medical insurance exist; also the rate of unplanned emergency dialysis was very high. All this emphasizes the need for urgent measures to reduce the incidence of ESRD in the country. This should focus on the prevention and treatment of risk factor especially diabetes, hypertension and infectious disease such as HIV. Sensitization, continue medical education and the implementation of a national program for prevention control of risk factors for CKD and early detection of CKD are some measure to undertake.

## Abbreviations

ANOVA: Analysis of the variance
BP: Blood pressure
CKD: Chronic kidney disease
DGH: Douala General Hospital
eGFR: Estimated glomerular filtration rate
ERSD: End-stage renal disease
HD: Haemodialysis
HIV: Human immunodeficiency virus
RRT: Renal replacement therapy
SD: Standard deviation
SSA: Sub-Saharan Africa
